# EGR1 Regulates Transcription Downstream of Mechanical Signals during Tendon Formation and Healing

**DOI:** 10.1371/journal.pone.0166237

**Published:** 2016-11-07

**Authors:** Ludovic Gaut, Nicolas Robert, Antony Delalande, Marie-Ange Bonnin, Chantal Pichon, Delphine Duprez

**Affiliations:** 1 Sorbonne Universités, UPMC Univ Paris 06, CNRS UMR7622, Inserm U1156, IBPS-Developmental Biology Laboratory, F-75005 Paris, France; 2 CNRS UPR4301-CBM, 45071 rue Charles Sadron, Orléans CEDEX2, France; Mayo Clinic Minnesota, UNITED STATES

## Abstract

**Background:**

Tendon is a mechanical tissue that transmits forces generated by muscle to bone in order to allow body motion. The molecular pathways that sense mechanical forces during tendon formation, homeostasis and repair are not known. EGR1 is a mechanosensitive transcription factor involved in tendon formation, homeostasis and repair. We hypothesized that EGR1 senses mechanical signals to promote tendon gene expression.

**Methodology/Principal findings:**

Using *in vitro* and *in vivo* models, we show that the expression of *Egr1* and tendon genes is downregulated in 3D-engineered tendons made of mesenchymal stem cells when tension is released as well as in tendon homeostasis and healing when mechanical signals are reduced. We further demonstrate that EGR1 overexpression prevents tendon gene downregulation in 3D-engineered tendons when tension is released. Lastly, ultrasound and microbubbles mediated EGR1 overexpression prevents the downregulation of tendon gene expression during tendon healing in reduced load conditions.

**Conclusion/Significance:**

These results show that *Egr1* expression is sensitive to mechanical signals in tendon cells. Moreover, EGR1 overexpression prevents the downregulation of tendon gene expression in the absence of mechanical signals in 3D-engineered tendons and tendon healing. These results show that EGR1 induces a transcriptional response downstream of mechanical signals in tendon cells and open new avenues to use EGR1 to promote tendon healing in reduced load conditions.

## Introduction

Tendon is a crucial component of the musculo-skeletal system, which transmits forces generated by skeletal muscle to bone to allow body motion. Mechanical signals are known to be involved in tendon development, homeostasis and repair [1–4]. However, the mechanotransduction pathways involved in tendon cell differentiation in normal or pathological situations are not fully understood.

The functional component of tendon is type I collagen, which displays a tendon-specific spatial organization that confers tendon mechanical properties [5]. Type I collagen is composed of a triple helix of collagen chains (α1(2), α2(1)) coded by two different genes, *Col1a1* and *Col1a2*. Unfortunately, neither of the *Col1a* genes is specific to tendons. They are also expressed in many other connective tissues, making it difficult to assess tenogenesis using *Col1a* gene expression. The bHLH transcription factor Scleraxis (Scx) is specifically expressed in embryonic, fetal and postnatal tendons [6–8]. From the 4^th^ postnatal month, *Scx* expression is restricted to the epitenon, but is reactivated in the tendon core by treadmill exercise [8]. Moreover, *Scx* expression is upregulated in tendons upon injury in animal models [9, 10]. The type II transmembrane glycoprotein tenomodulin (Tnmd) is also considered as a relevant marker of tendon cell differentiation in mouse, rat and human [11–13]. During development, *Scx* is required and sufficient for *Tnmd* expression in limb tendons [14, 15]. *Tnmd* ^-/-^ mice show defective postnatal tendons [16] and reduced self-renewal of tendon stem cells in adult [17].

In addition to *Scx*, the Zinc finger transcription factor, Early Growth Response-1 (EGR1) has been identified as being involved in pre- and postnatal tendon formation [10, 18]. EGR1 is not specific to tendons, since it is expressed in many other tissues; however, EGR1 has the remarkable ability to be sufficient for promoting tendon gene expression, including *Scx* and *Col1a1*, during development [18], in mouse mesenchymal stem cells [10] and in rabbit tendon stem cells [19]. The DNA-binding protein EGR1 is known to be a mechanosensitive gene in various cellular systems. Mechanical stretch increases *Egr1* transcription in endothelial cells [20], vascular smooth muscle cells [21] or skeletal muscle cells [22]. Dynamic compression also activates *Egr1* transcription in 3-dimensional (3D) cultures of mouse primary chondrocytes [23]. EGR1 has been shown to be a mediator of mechanical input that contributes to vascular remodeling of vein grafts [24].

Since EGR1 is known to be a mechanosensitive gene and involved in tendon development, homeostasis and repair, we hypothesized that EGR1 transduces mechanical signals into transcriptional regulation to promote tendon cell differentiation during tendon formation, homeostasis and repair. We used *in vitro* 3-dimensional culture system and *in vivo* models to test this hypothesis.

## Materials and Methods

### Animals

The *Egr1*^*LacZ/+*^ mice bred in a C57BL/6j background carry an insertion of a LacZ-neo cassette that inactivates the *Egr1* gene [25] and allow the visualization of *Egr1* expression with LacZ activity in a heterozygous context [10]. C57BLj wild-type mice were purchased from Janvier (France). All animal experiments were conducted in accordance with the guidelines of the french national ethic comity for animal experimentation N°05. The animal experiments shown in this study have been approved by the french national ethic committee for animal experimentation N°05 and are registered under the number 01789.02.

### Engineered tendons made of mesenchymal stem cells

Mouse mesenchymal stem cells, C3H10T1/2 [26] were used to establish fibrin-based 3D constructs. Tendon-like structures from mouse C3H10T1/2 cells or C3H10T1/2-EGR1 cells [10] were performed as previously described [27]. For each construct, 400 μl of cell suspension (7.5 10^5^ cells) were mixed with 20 mg/ml fibrinogen (Sigma, St Louis, MO, USA) and 200 U/ml thrombin (Sigma, St Louis, MO, USA). The fibrin gels containing cells were seeded in already prepared SYLGARD-covered wells (Dow Chemical, Midland, MI, USA), in which two 8 mm-sutures (Ethican, Sommerville, NJ, USA) were pinned 10 mm apart. Culture medium containing 200 μM of L-ascorbic acid 2-phosphate was added to the wells and gels were scored every day for a proper contraction into a linear construct. After 7 days, the C3H10T1/2 and C3H10T1/2-EGR1 cells formed continuous tendon-like constructs between the 2 anchors. Tension release was obtained by cutting one end of the construct as previously described in [28]. Gene expression was analyzed 24 hours after tension release. Each tendon construct made of C3H10T1/2 or C3H10T1/2-EGR1 cells under tension or after tension release was considered as a biological sample. We analyzed 12 constructs made of C3H10T1/2 cells, 7 constructs made of C3H10T1/2-EGR1 cells, 7 de-tensioned-constructs made of C3H10T1/2 cells and 5 de-tensioned-constructs made of C3H10T1/2-EGR1 cells. The mRNA levels of each construct were analyzed by q-RT-PCR.

### Botox injection in muscles and Achilles tendon injury in adult mice

For the analysis of mechanical signal involvement in tendon homeostasis, 6 UI/kg of Botox preparation (Allergan) [29] and physiological saline solution were injected into the Gastrocnemius muscles of right and left legs, respectively, of *Egr1*^*LacZ/+*^ (N = 6) and wild-type (N = 12) adult mice (two- to four-month-old). Botox injection reduces muscle contraction and movements and consequently mechanical signals to tendons [30]. Experimental animals did not display any obvious difficulty in moving, but limped on the Botox-injected legs. The Botox and physiological saline manipulated *Egr1*^*LacZ/+*^ animals were kept for one week (N = 6) and analyzed for LacZ staining. Tendons were fixed for 20 minutes in 4% paraformaldehyde and incubated in X-gal staining solution for 4 hours at 37°C. The Botox and physiological saline manipulated wild-type animals were kept for one (N = 8) or two (N = 11) weeks after injection (3 independent experiments) and then analyzed for gene expression.

For the analysis of mechanical signal involvement in tendon healing, Achilles tendon injury was performed on left legs of wild-type animals, as previously described in [10]. Briefly, adult mice were anesthetized by isoflurane inhalation. A 0.5-mm longitudinal full-thickness lesion parallel to the axis of the tendon was performed using a scalpel. In this type of injury, the tendon tension was maintained. After injury, the skin was sutured using 2–0 Mersilk, and the animals were kept for two weeks. The Botox or physiological saline was injected in the Gastrocnemius muscles of the manipulated left legs. The mouse group, which had undergone Achilles tendon injury and Botox injection, constitute the experimental group (N = 11) that was compared to the control group with Achilles tendon injury and physiological saline injection in muscles (N = 10). Experimental animals did not display any obvious difficulty in moving. However, the animals in the group that had Achilles tendon injury and Botox injection were limping compared to those in the group with Achilles tendon injury and physiological saline injection. Achilles tendons, in the above experimental conditions, were processed for RT-q-PCR analyzes.

### Ultrasound and microbubbles-mediated EGR1 gene delivery in Achilles tendons followed by tendon injury and Botox injection into muscles

Ultrasound and microbubbles-mediated gene delivery is a technique also known as sonoporation, allowing for a high and sustained gene expression in mouse Achilles tendons [31]. 10 μg of EGR1 encoding plasmid DNA [18, 32] and 3.75 μl of MicroMarker^TM^ microbubbles (Bracco) in a total volume of 10 μl were injected into the Achilles tendon. Injection was immediately followed by a 1-MHz ultrasound stimulation of 200 kPa (negative peak) during 10 minutes. Ultrasound was generated from a 0.5" diameter, IBMF-014 transducer with a frequency of 1 MHz (Sofranel, Sartrouville, France). A signal consisting of 40 cycles with a frequency of 1.0 MHz and a pulse repetition frequency of 10 kHz, a duty cycle of 40%, was generated by a 33220A arbitrary function generator (Agilent technologies, Les Ulis, France). The signal was amplified by a RF power amplifier (ADECE, Artannes, France) and used as the input for the transducer. The transducer was calibrated in a Perspex container using an HGL-200 PVDF bullet type hydrophone (Onda, Sunnyvale, CA). EGR1 sonoporation experiments were performed one day before tendon injury and Botox treatment because intratendinous injection requires the integrity of tendon sheath. Mice were euthanatized 15 days after tendon injury and Botox injection and tendons were harvested in 500 μl of RNAlater solution (ThermoScientific) for RNA isolation and RT-q-PCR analyses. 10 mice displaying EGR1 overexpression, tendon injury and Botox injection and 6 mice displaying tendon injury and Botox injection were analyzed.

### RNA isolation, reverse transcription and RT-q-PCR

Total RNAs were extracted from fibrin gel constructs made of C3H10T1/2 (N = 11) or C3H10T1/2-EGR1 cells (N = 7), tension-released fibrin gel constructs made of C3H10T1/2 cells (N = 7) or C3H10T1/2-EGR1 cells (N = 5). Total RNAs were extracted from adult mouse tendons under various experimental designs (following physiological saline or Botox injection in normal or injured conditions, after ultrasound-forced EGR1 expression followed by injury and Botox injection) according to the Qiagen RNeasy Minikit (QIAGEN, Germany). RNA (300 ng to 1 μg) was reverse transcribed using the High Capacity Retrotranscription Kit (Applied Biosystems). RT-q-PCR was performed using SYBR Green PCR Master Mix (Applied Biosystems). Relative mRNA levels were calculated using the 2^−ΔΔCt^ method [33]. In all our experimental designs, the Cts of two housekeeping genes *Gapdh* and *Hprt* did not show any variations in control versus experimental conditions. The ΔCts were obtained from Cts normalized with *Gapdh* levels in each sample. RNA samples originating from 5 to 11 biological samples originating from 3 independent experiments were analyzed in duplicate. Primers used for RT-q-PCR are listed in Table 1.

**Table 1:** Primers used for quantitative RT-PCR.

Col1a1	Fwd 5’ CCAGCGAAGAACTCATACAGC
	Rev 5’ GGACACCCCTTCTACGTTGT
Col1a2	Fwd 5’ CCAGCGAAGAACTCATACAGC
	Rev 5’ GGACACCCCTTCTACGTTGT
Egr1	Fwd 5′-CAGCGCCTTCAATCCTCAAG
	Rev 5′-GCGATGTCAGAAAAGGACTCTGT
Gapdh	Fwd 5’ TTGTGGAAGGGCTCATGACC
	Rev 5’ TCTTCTGGGTGGCAGTGATG
Hprt	Fwd 5’AGGGCATATCCAACAACAAACTT
	Rev 5’GTTAAGCAGTACAGCCCCAAA
Scx	Fwd 5’ CCTTCTGCCTCAGCAACCAG
	Rev 5’ GGTCCAAAGTGGGGCTCTCCGTGACT
Tgfb2	Fwd 5’ GAATAAAAGCGAAGAGCTCGAGG
	Rev 5’ GAGGTGCCATCAATACCTGCA
Tnmd	Fwd 5’ AACACTTCTGGCCCGAGGTAT
	Rev 5’ AAGTGTGCTCCATGTCATAGGTTTT

### Statistical analysis

Sample sizes were based on historical experience of effect sizes for these types of experiments. Error bars in graphics represent the standard deviations or the standard errors of the mean, depending of the size of the samples. Statistics were calculated using non-parametric tests with the GraphPad Prism V6 software. The Mann-Whitney test was used for unpaired samples and the Wilcoxon test for paired samples.

## Results

### EGR1 overexpression prevents the decrease of tendon gene expression in 3D tendon constructs after tension release

**Fig 1:**
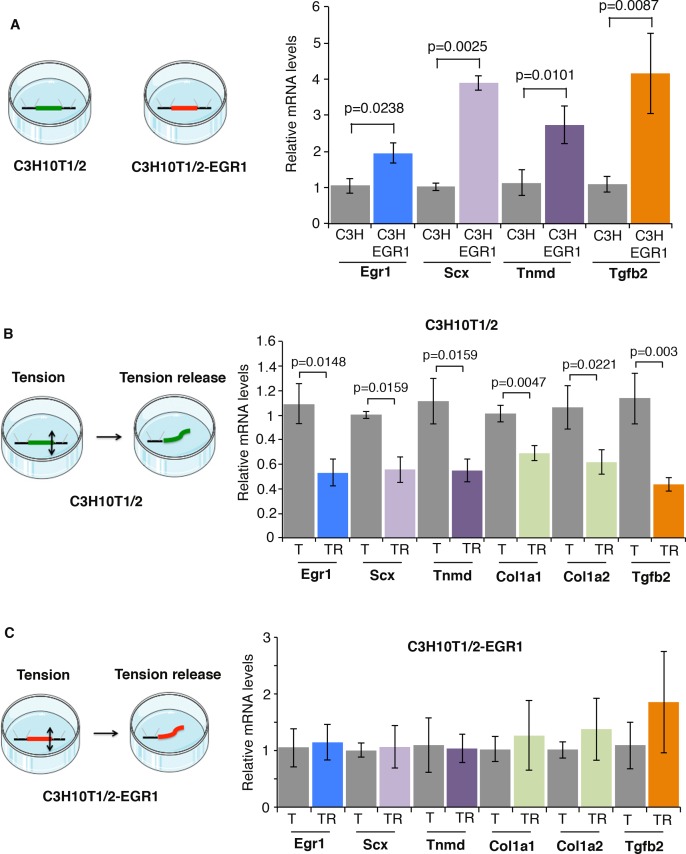
EGR1 overexpression prevents the downregulation of tendon-associated gene expression in tension released engineered tendons. (**A**) Two week-old fibrin gel constructs made of mouse C3H10T1/2 cells or C3H10T1/2-EGR1 cells were analyzed for tendon gene expression by RT-q-PCR analyses. The mRNA levels of C3H10T1/2 constructs were normalized to 1. Errors bars represent standard errors of the mean of 5 C3HT101/2 constructs and 7 C3H10T1/2-EGR1 constructs. The *p* values were calculated using the Mann-Whitney test. The mRNA levels of *Egr1*, *Scx*, *Tnmd* and *Tgfb2* genes were increased in C3H10T1/2-EGR1 constructs compared to those of C3H10T1/2 constructs. (**B**) Tension was released in C3H10T1/2 constructs by sectioning one end of the construct. Transcript levels were analyzed by RT-q-PCR analyses in tension-released C3H10T1/2 constructs and compared to C3H10T1/2 constructs. The mRNA levels of C3H10T1/2 constructs were normalized to 1. Errors bars represent standard errors of the mean of 7 C3H10T1/2 constructs and 7 tension-released C3H10T1/2 constructs. The *p* values were calculated using the Mann-Whitney test. We observed a decrease in the transcript levels of *Egr1*, *Scx*, *Tnmd*, *Col1a1*, *Col1a2* and *Tgfb2* genes in tension-released C3H10T1/2 constructs (TR) compared to tensioned C3H10T1/2 constructs (T). (**C**) The mRNA levels of tendon genes were analyzed in tension-released C3H10T1/2-EGR1 constructs and compared with tensioned C3H10T1/2-EGR1 constructs by RT-q-PCR analyses. The mRNA levels of C3H10T1/2-EGR1 constructs were normalized to 1. Errors bars represent standard errors of the mean of 7 C3HT101/2-EGR1 constructs and 5 tension-released C3H10T1/2-EGR1 constructs. The *p* values were calculated using the Mann-Whitney test. There was no significant change in tendon gene expression in tension-released C3H10T1/2-EGR1 constructs (TR) compared to tensioned C3H10T1/2-EGR1 constructs (T). T, Tension, TR, Tension release.

Fibrin-based 3D cell cultures recapitulate tendon formation based on tenogenic marker expression and tendon-like collagen fibrillogenesis [27, 34–37]. This *in vitro* engineered tendon system involves tension [34, 35]. Forced expression of EGR1 in fibrin-based 3D C3H10T1/2 cell cultures increases *Scx*, *Col1a1* and *Col1a2* expression levels compared to control fibrin-based 3D C3H10T1/2 cell cultures [10]. We now show that the expression of the tendon differentiation marker *Tnmd* was also increased in the presence of EGR1 in 3D constructs (Fig 1A). The *Tgfb2* mRNA levels were also increased in EGR1-producing 3D constructs compared to 3D constructs (Fig 1A). The tension release of the 3D constructs induces the appearance of immature collagen fibrils with no preferred orientation in engineered chick tendons [27] and loss of *TNMD* expression in engineered human tendons [28]. The tension was released by cutting one edge of the engineered mouse tendons made of C3H10T1/2 cells (Fig 1B). Tension release led to a decrease in the expression of *Egr1* and tendon genes including *Scx*, *Tnmd*, *Col1a1* and *Col1a2* (Fig 1B). The expression of *Tgfb2* was also decreased in tension-released engineered tendons (Fig 1B). Tension release in Egr1-producing 3D-constructs did not trigger any significant changes in tendon gene expression compared to Egr1-producing 3D constructs under tension (Fig 1C). This shows that EGR1 overexpression is able to activate tendon gene expression independently of tension in engineered tendons. We conclude that *Egr1* expression is sensitive to tension in engineered mouse tendons and EGR1 forced expression prevents the downregulation of tendon gene expression in the absence of mechanical input.

### The expression of tendon genes is downregulated in adult tendons when movements are reduced

**Fig 2:**
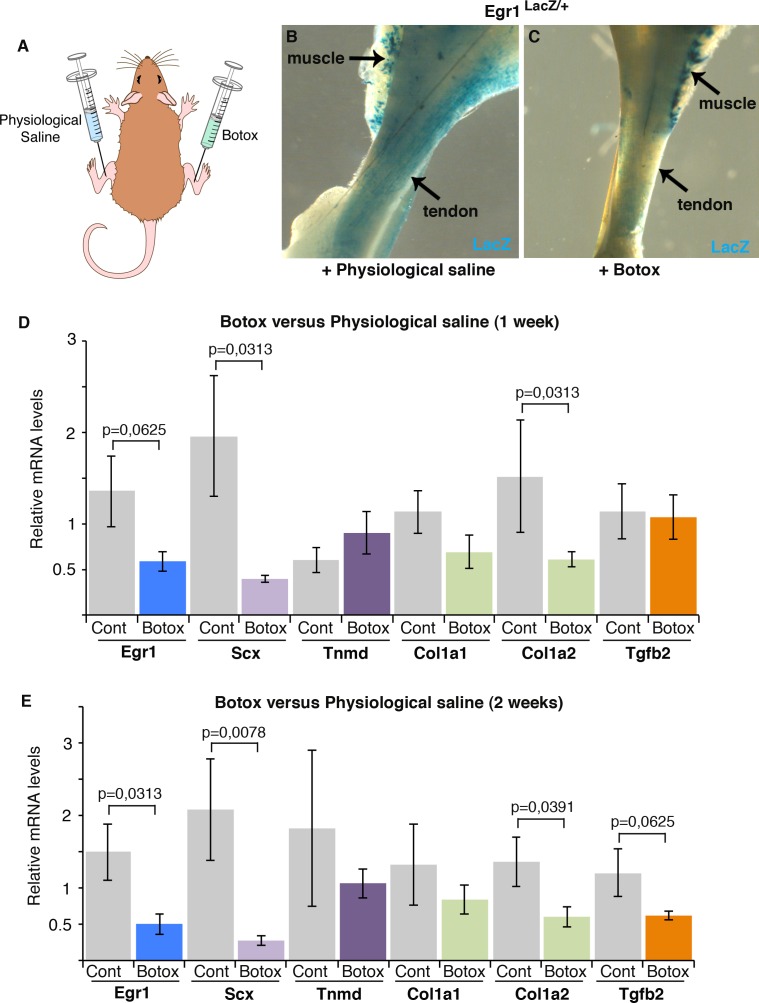
Reduced mechanical input induces a diminution of *Egr1* and *Scx* expression in adult tendons. (**A**) Botox or physiological saline injections in Gastrocnemius muscles of adult mice. (**B,C**) LacZ staining (reflecting Egr1 expression) in tendons, 1 week following physiological saline or Botox injection in Egr1^Lacz/+^ adult mice. (**D**) RT-q-PCR analyses of tendons, one or two weeks after Botox or physiological saline injections in muscles in adult mice. The mRNA levels of tendons following Botox injection were compared to those of tendons with physiological saline injection. Errors bars represent the standard deviations of 5 (one week) and 7 (two weeks) biological samples. The mRNA levels of *Egr1*, *Scx*, *Col1a2*, *Tgfb2* genes were decreased one or two weeks after Botox injection compared to physiological saline injection. *Tnmd* mRNA levels were not significantly decreased after Botox injection. The *p* values were calculated using the Wilcoxon test.

In order to assess the importance of mechanical signals for tendon gene expression in homeostasis, we developed a reduced load model in adult mice based on botulinium toxin A (Botox) injection into the Gastrocnemius muscle of hindlimbs (Fig 2A). One week after injection of Botox or physiological saline solution in muscles of *Egr1*^*LacZ/+*^ mice, LacZ expression (reflecting Egr1 expression) appeared to be decreased in Achilles tendons (Fig 2B and 2C). Consistently, the *Egr1* mRNA levels were decreased in Achilles tendons of Botox-injected legs compared to saline solution-injected legs, one and two weeks after injection in wild-type mice (Fig 2D). *Scx* expression was also decreased in tendons of Botox-injected legs compared to control legs, one and two weeks after Botox injection (Fig 2D), consistent with the decrease in *Scx* expression one week after Botox injection in Scx-GFP mice [30]. The tendon-associated collagen gene *Col1a2* also displayed decreased expression levels one and two weeks after Botox injection (Fig 2D). The expression of the terminal differentiation tendon marker, *Tnmd* did not significantly change (Fig 2D). Interestingly, *Tgfb2* expression was also decreased in tendons, two weeks after Botox injection (Fig 2D). This showed that the expression of *Egr1*, *Tgfb2* and tendon genes (*Scx* and *Col1a2*) is sensitive to mechanical input in adult tendons. We conclude that mechanical signals are required for tendon gene expression in homeostasis.

### Reduced mechanical signals decrease the transcriptional response during tendon healing after injury

**Fig 3:**
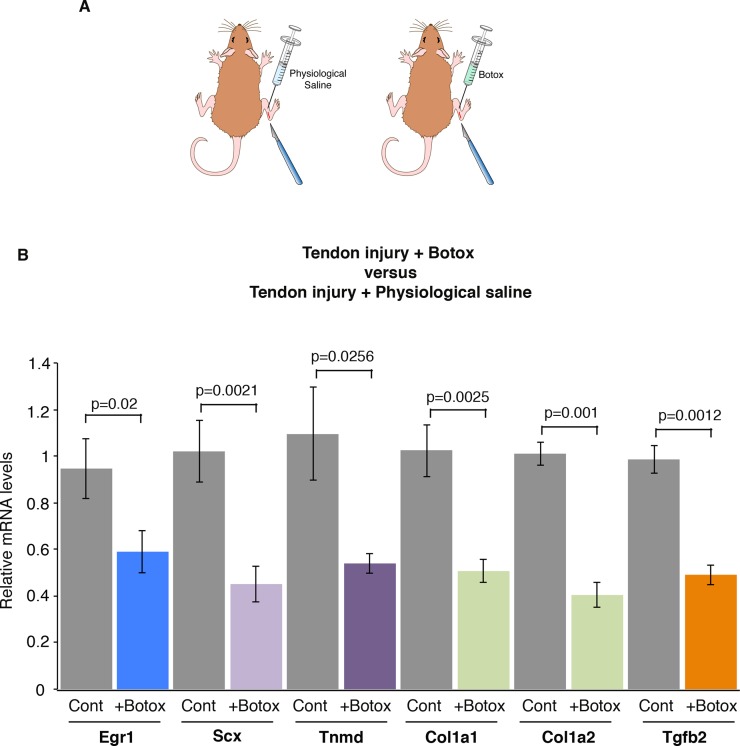
Mechanical signals are required for normal tendon gene response following tendon injury. **(A)** Botox or physiological saline injections in Gastrocnemius muscles and tendon injury in adult mice. (**B**) RT-q-PCR analyses of tendons, 2 weeks after tendon injury and Botox injection in muscles. The mRNA levels of tendons following injury and physiological saline injection in muscles were normalized to 1. The errors bars represent the standard error of the means of 10 and 11 biological samples of injured tendons after physiological saline or Botox injections, respectively. The *p* values were calculated using the Mann-Whitney test. The mRNA levels of the *Egr1*, *Scx*, *Tnmd*, *Col1a1*, *Col1a2* and *Tgfb2* genes were all significantly decreased in reduced movement conditions compared to controls during the healing process, following tendon injury.

In order to analyze the effect of mechanical signals during healing, we used an Achilles tendon injury model in adult wild-type mice as previously described [10]. In this tendon injury model, a longitudinal incision was performed along the axis of the Achilles tendon, in which the tension was maintained. One week after tendon injury, there is a dramatic increase in tendon gene expression, including *Scx*, *Tnmd*, *Col1a1* and *Col1a2* [10]. *Egr1* expression is also increased by fold-3.6 in tendons, after injury and *Egr1* is required for the normal tendon transcriptional response during the healing process [10]. In order to determine whether mechanical signals would influence the transcriptional response during tendon healing, we injected Botox or physiological saline solution in muscles in this mouse model of tendon injury (Fig 3A). We observed a significant decrease in the expression of *Egr1*, *Scx*, *Tnmd*, *Col1a1*, *Col1a2* and *Tgfb2* genes. after Botox injection compared to physiological saline solution injections, in injury conditions (Fig 3B). The decrease of mRNA levels of tendon genes ranged from 40% to 60% (Fig 3). This shows that a diminution of mechanical signals modifies the transcriptional response during the tendon healing process following injury. We conclude that mechanical signals are required for the full transcriptional response during tendon healing.

### EGR1 forced expression in tendons prevents the diminution of tendon gene response in reduced load conditions after injury

**Fig 4:**
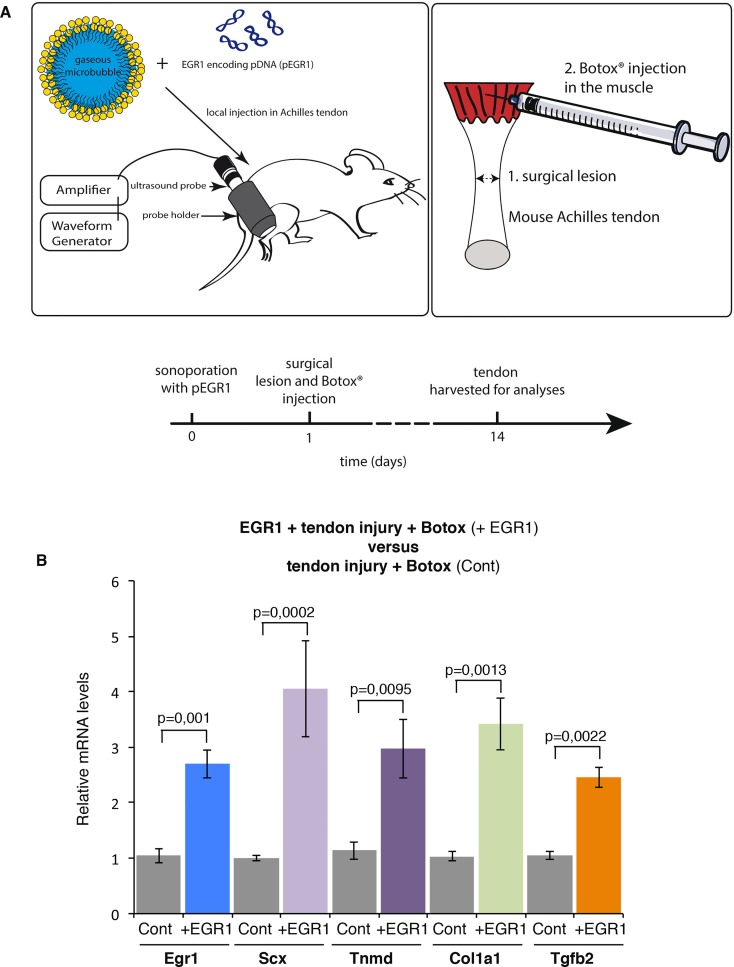
EGR1 forced expression in tendons prevents the diminution of tendon gene expression during tendon healing in reduced mechanical load. (**A**) Description of the experimental design for sonoporation. 10 μg of EGR1 encoding plasmid and 3.75 μl of MicroMarker microbubbles were injected in the Achilles tendon sheath. Tendons were then stimulated by ultrasound at 1 MHz during 10 minutes at 200 kPa, 40% duty cycle and 10 kHz pulse repeating frequency. The day after EGR1 sonoporation, a surgical lesion of the Achilles tendon was performed followed by a Botox or physiological saline solution injection in the muscle. Two weeks after treatment, tendons were harvested for analyses by RT-q-PCR. (**B**) RT-q-PCR analysis of tendon gene expression in EGR1-sonoporated tendons versus control-tendons, following tendon injury in immobilization conditions. The mRNA levels of control tendons following injury and Botox injection in muscles were normalized to 1. The error bars represent standard errors of the mean of 6 biological samples of injured tendons of Botox-injected legs in the absence of EGR1 and 10 biological samples of injured tendons of Botox-injected legs in presence of ectopic EGR1. The *p* values were calculated using the Mann-Whitney test. The mRNA levels of *Egr1*, *Scx*, *Tnmd*, *Col1a2* and *Tgfb2* were increased in Egr1-sonoporated tendons compared to control tendons, following tendon injury and Botox injection in muscles.

The expression of the mechanosensitive gene *Egr1* was decreased in reduced load conditions in tendon homeostasis (Fig 2) and healing (Fig 3). To assess whether EGR1 acts downstream of mechanical signals during tendon healing, we performed *in vivo* EGR1 rescue experiments in reduced load conditions and after tendon injury. We took advantage of an ultrasound-based gene delivery method, which has been found to be efficient in tendons [31]. Plasmid DNA encoding for the *Egr1* gene was delivered to the tendons with optimized parameters (1 MHz, 200 kPa, 10 min, 40% duty cycle and 10 kHz pulse repetition frequency). The following day, Botox was injected in muscle followed by tendon injury (Fig 4A). Two weeks after Botox injection in muscle and tendon injury, we compared the expression of tendon genes in tendons overexpressing EGR1 or not during tendon healing and in reduced load conditions. In this experiment, *Egr1* was increased by fold-2.6. EGR1 forced expression in tendons increased the expression of tendon-associated genes including *Scx*, *Tnmd*, *Col1a1*, *Col1a2* and *Tgfb2*, when compared to control (Fig 4B). The levels of induction were similar to that of *Egr1* overexpression (Fig 4A). This shows that EGR1 is sufficient to prevent the diminution of tendon gene expression that is observed during healing in reduced load conditions.

## Discussion

We show that reduced load conditions consistently lead to a decrease in expression of the transcription factor *Egr1* and tendon genes in tendon homeostasis, tendon healing and *in vitro* engineered tendons. We also demonstrate that EGR1 forced expression prevents the decrease of tendon gene expression in reduced load conditions during tendon healing and in engineered tendons.

*Egr1* is a mechanosensitive gene, which has been shown to act downstream of mechanical signals in the vascular system [20, 21, 24]. Consistently, *Egr1* expression is systematically downregulated in reduced load conditions in the *in vivo* and *in vitro* models of tendon biology. The decrease of *Egr1* expression at the transcription level in reduced load conditions is consistent with the upregulation of *Egr1* expression in overload conditions in adult tendons [38, 39]. *Egr1* expression is induced as early as 15 minutes after a loading episode in rat Achilles tendons [40]. This indicates that *Egr1* expression reflects a rapid transcriptional response following loading changes in tendons. In addition to being sensitive to mechanical signals, *Egr1* appears to be sufficient to drive the tendon genetic program in the absence of mechanical signals. The presence of exogenous EGR1 abolishes the downregulation of *Scx*, *Col1a1* and *Tnmd* in engineered tendons subjected to tension release and EGR1 also prevents the decrease of *Scx*, *Col1a1* and *Tnmd* expression in Achilles tendons in reduced load conditions during tendon healing following injury. This shows that the Zinc-finger transcription factor *Egr1* senses mechanical signals in tendons and modulates tendon gene expression upon loading changes. Although EGR1 has the ability to induce the expression of tendon genes in normal [10] or reduced load (Figs 1 and 4) conditions, it remains unclear whether EGR1 directly regulates the transcription of tendon genes. Previous promoter and Chromatin Immuno-Precipitation (ChIP) analyses indicate that EGR1 trans-activates the tendon promoter of the mouse *Col1a1* gene [18, 32]. Moreover, EGR1 directly regulates *Tgfb2* transcription in adult mouse tendons [10]. *Tgfb2* expression is downregulated in reduced load conditions in tendon homeostasis or healing in adult mice (Figs 2 and 3) and in engineered tendons made of mouse mesenchymal stem cells (Fig 1). EGR1 rescue experiments in conditions of reduced mechanical signals induced *Tgfb2* expression in engineered tendons and during the tendon healing process (Figs 1 and 4). These results suggest that *Egr1* acts upstream of *Tgfb2* to activate tendon gene expression upon loading. It should be noted that TGF-ß1 supplementation was not sufficient to prevent the decrease of tendon gene expression in de-tensioned 3D engineered tendons derived from human tendon cells [28]. It is not clear whether TGF-ß ligand is not sufficient to activate tendon gene expression in reduced load conditions or TGF-ß1 activity differs from TGF-ß2 activity. Antagonist effects between TGF-ß1 and TGF-ß2 ligands have been reported on *COL1A1* transcription in rat tendon fibroblasts [41]. TGF-ß supplementation activates *Scx* expression in various cellular models and loss of TGF-ß leads to a decrease of *Scx* expression in developmental tendons [10, 42–44]. We failed to identify any EGR1 binding sites in mouse *Scx* promoter region and all our ChIP attempts to identify direct EGR1 recruitment to *Scx* promoter region were unsuccessful. Given the EGR1 direct binding to *Tgfb2* promoter [10], it is tempting to suggest that TGF-ß2 mediates the *Scx* induction by EGR1 in tendons. Consistently, SCX and SMAD3 (an intracellular component of the TGF-ß pathway) physically interact in 10T1/2 cells [45] and SCX is required for Smad3-mediated gene transcription in cardiac fibroblasts [46]. Whether EGR1 activates *Tnmd* via TGF-ß2 is less clear, since TGF-ß2 has been shown to downregulate *Tnmd* expression in mouse stem cells cultured in 2D [10, 44, 47] and do not have any positive effect on *TNMD* expression in human 3D tissue cultures [28]. In contrast, SCX gain- and loss-of-function experiments lead to upregulation and downregulation of *Tnmd*, respectively, during development [14, 15]. This suggests that SCX could activate *Tnmd* expression during tendon differentiation.

**Fig 5:**
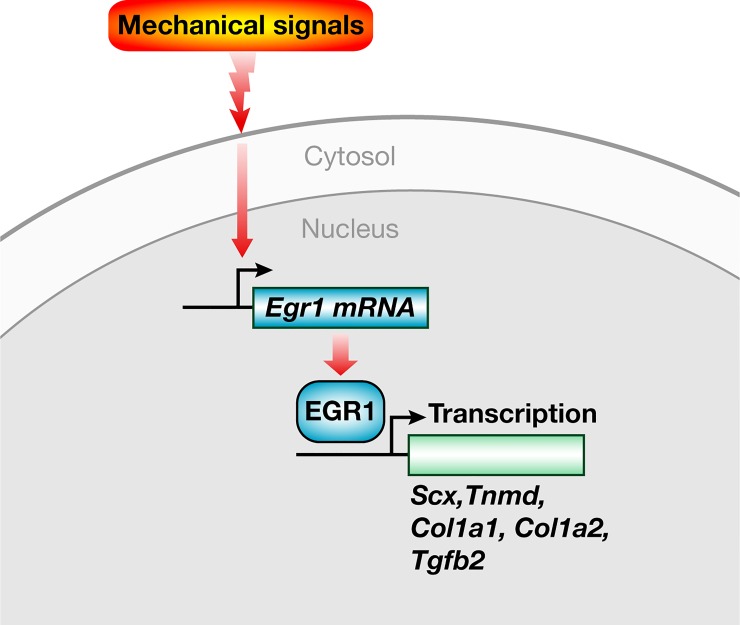
Schematic representation of EGR1 regulation and function downstream of mechanical signals. *Egr1* expression is regulated by mechanical signals in tendon cells. EGR1 positively regulates the transcription of tendon genes including *Scx*, *Tnmd*, *Col1a1* and *Co1a2*. The transcription of *Tgfb2* is also regulated by EGR1.

In summary, we show that the expression of the zinc-finger transcription factor EGR1 is sensitive to mechanical signals and that EGR1 senses mechanical signals at the transcription level. Moreover, EGR1 is sufficient to promote tendon gene expression in the absence of mechanical forces during tendon formation and healing (Fig 5). This feature opens the possibility of exploiting EGR1 forced expression to accelerate tendon healing in reduced load conditions.
